# Assessing the degree of stratification between closely related Holstein-Friesian populations

**DOI:** 10.1007/s13353-017-0409-2

**Published:** 2017-10-06

**Authors:** Joanna Szyda, Tomasz Suchocki, Saber Qanbari, Zengting Liu, Henner Simianer

**Affiliations:** 1 Biostatistics Group, Department of Genetics, Wrocław University of Environmental and Life Sciences, Kozuchowska 7, 51-631 Wrocław, Poland; 2 National Research Institute of Animal Production, Krakowska 1, 32-083 Balice, Poland; 3 Animal Breeding and Genetics Group, Georg-August-Universität Göttingen, Albrecht-Thaer-Weg 3, 37075 Göttingen, Germany; 4 vit, Heinrich-Schröder-Weg 1, 27283 Verden, Germany

**Keywords:** German and Polish Holstein-Friesian cattle, Linkage disequilibrium, Production traits, Single nucleotide polymorphism, Somatic cell score

## Abstract

Genomic information is an important part of the routine evaluation of dairy cattle and provides the wide availability of animals genotyped using single nucleotide polymorphism (SNP) microarrays. We analyzed 2243 Polish and 2294 German Holstein-Friesian bulls genotyped using the Illumina BovineSNP50 BeadChip. For each bull, estimated breeding values (EBVs) calculated from national routine genetic evaluation were available for production traits and for somatic cell score (SCS). Separately for each population, we estimated SNP haplotypes, pairwise linkage disequilibrium (LD), and SNP effects. The SNP genetic covariance between both populations was estimated using a bivariate mixed model. The average LD was lower in the Polish than in the German population and, with increasing genomic distance, LD decays 1.7 times more rapidly in German than in Polish cattle. The comparison of SNP allele frequencies for base populations estimated separately using Polish and German data revealed a very good agreement. The comparison of genetic effects corresponding to various window lengths defined in bp emerged a systematic pattern: regardless of the length of the compared region, few significant differences were found for production traits, while many were observed for SCS. For each trait, the German population had much higher SNP variances than the Polish population and the genetic covariance estimates were all positive. Depending on traits’ inheritance mode, the additive genetic variation can be stored in many genes following the infinitesimal model (like for SCS) or distributed between genes with high effects and the polygenic “background” (like for production traits). Accounting for those differences has implications on the prospective international genomic evaluation.

## Introduction

In dairy cattle, many countries have incorporated genomic information into their genetic evaluation systems (Hayes et al. 2009; VanRaden et al. 2009) and it has become evident that the genomic information is now an important part of the routine evaluation of genetic merit of dairy cattle (Liu et al. 2010). From 2006 onwards, such programs have also been implemented in Germany and Poland.

Apart from conducting genomic evaluation on an industrial basis, such data is also a valuable source of information for geneticists, since the broad availability of single nucleotide polymorphisms (SNPs) genotyped on well-defined populations with detailed information on phenotypes, environmental factors, and pedigree provides a basis for investigating the genetic background of complex traits. Moreover, a substantial number of genome-wide association studies (GWAS) has been performed on traits routinely measured and selected in dairy cattle, which is best illustrated by 34,754 QTL and association results of 731 studies submitted to the cattleQTLdb (http://www.animalgenome.org/QTLdb/cattle, release 31). As a consequence, several genes with large effects on such traits have been identified as candidate genes (e.g., PPARGC1 and APBB2, Suchocki et al. 2013) or even in the form of causal mutations (e.g., DGAT1, Grisart et al. 2002). Currently, in genetic analyses of dairy populations, the emphasis is put on genes with intermediate additive effects and on loci of which their impact on trait variation is manifested through non-additive effects, such as dominance or epistasis (e.g., Sun et al. 2014; Kemper et al. 2016). The former are of great interest in selection programs, especially in view of the possible decrease of genetic variation attributed to major genes, while the latter are important for a better understanding of the genetics of complex traits. Unfortunately, in order to guarantee reasonable type 1 and (especially) type 2 error rates in hypothesis testing involving such genes, a large sample size is required. On one hand, very large sample sizes are currently relatively easy to obtain for dairy cattle thanks to the common use of the Illumina BovineSNP50 BeadChip in national selection programs and exchange of data between countries. On the other hand, national populations may differ in selection goals and, consequently, in biological adaptation as a response to selection, which, furthermore, affects SNP allele frequencies and linkage disequilibrium (LD) patterns (Rosenberg et al. 2010). For instance, in the German Holstein-Friesian population, selection has been based on a balanced breeding goal comprising production, reproduction, and functional traits, while in the Polish population, for many decades, the breeding emphasis had been solely put on protein and fat yields, with other, non-production, traits included in the selection index only from 2007, and a functional longevity from 2014. Therefore, it is essential to know the differences in the genomic structure of populations before considering country-specific data in a joint analysis.

The aim of the study was to use estimated breeding values (EBVs) and SNP genotypes to compare the patterns of genetic diversity between German and Polish Holstein-Friesian populations. In particular, the investigated aspects cover: (1) the comparison of LD patterns, (2) the assessment of differences in the effects of genomic regions on the selected traits, (3) the evaluation of differences in SNP allele frequencies, and (4) the estimation of the polygenic (co)variance components for production and udder health traits between German and Polish Holstein-Friesian populations.

## Materials and methods

### Dataset

The analyzed datasets comprised 2243 Polish and 2294 German Holstein-Friesian bulls. Both groups were defined as bulls for which EBVs on a national basis were available only for one of the countries. For each bull, EBVs calculated based on the national routine genetic evaluation models and corresponding effective daughter contributions were available for milk (MY), protein (PY), and fat (FY) yields, as well as for somatic cell score (SCS). Moreover, each individual was genotyped using the Illumina BovineSNP50 BeadChip version 1. Separately for each dataset, SNPs were filtered based on two criteria: the minor allele frequency (MAF) had to be ≥ 0.01 and the call rate ≥ 90%. Only SNPs that were present in both populations after filtering were kept, resulting in a final list comprising 39,557 SNPs representing an intersection of markers in the German and Polish datasets.

### Estimation of linkage disequilibrium

SNP haplotypes were estimated separately for both populations using fastPHASE (Scheet and Stephens 2006). The *r* ^2^ statistic (Hill and Weir 1994) estimated by Haploview (Barrett et al. 2005) was used to quantify LD between pairs of linked SNPs:

$$ {r}^2=\frac{D^2}{p_1\left(1-{p}_1\right){p}_2\left(1-{p}_2\right)}\kern0.5em , $$

where *D* is the deviation from LD and *p*_*i*_ represents the minor allele frequency of the 1st and 2nd of the compared SNPs. LD was estimated separately for Polish and German populations considering a set of SNP pairs located within the following Mbp intervals: [0–0.025), [0.025–0.05), [0.05–0.075), [0.075–0.12), [0.12–0.2), [0.2–0.5), [0.5–1.5), [1.5–3), and [3–5]. In order to estimate the shape of LD decay in each population, the regression function ${r}_{ix}^2={\alpha}_x+{\beta}_x\frac{1}{\sqrt{d_i}}+{e}_{ix}$ was fitted to the data, where subscript *x* represents the Polish (*x* = PL) or German (*x* = DE) population, ${r}_{ix}^2$ is the LD using the above formula averaged for SNPs within each distance interval *i*, and *d* represents the upper boundary of this interval. Paired *t*-tests were used to test for differences in the average LD between the Polish and German populations (*D*_PL, DE_) at a given Mbp interval, with the underlying* H*_0_:*D*_PL, DE_ = 0.

### Estimation of SNP effects

SNP effects were estimated separately for both datasets, using:

$$ \mathbf{y}=\mathbf{Xb}+\mathbf{Zg}+\mathbf{e}, $$

where **y** represents a vector of deregressed conventional breeding values of bulls, **X** is a design vector for fixed effects, **b** is a vector of fixed effects, which, here, comprises a general mean only, **Z** is a design matrix for SNP genotypes, which is parameterized as − 1, 0, or 1 for a homozygous, a heterozygous, and an alternative homozygous genotype, respectively, **g** is a vector of random additive SNP effects, and **e** is a vector of residuals. The covariance structure of the model comprises $\mathbf{g}\sim N\left(0,\mathbf{I}\frac{{\widehat{\sigma}}_a^2}{39557}\right)$, with **I** being an identity matrix and ${\widehat{\sigma}}_a^2$ representing the estimate of total additive genetic variance of a given trait provided by the routine genetic evaluation system for the whole active population of Polish and German Holstein-Friesian cattle, respectively, and $\mathbf{e}\sim N\left(0,\mathbf{D}{\sigma}_e^2\right)$, with **D** being a diagonal matrix of the reciprocal of the effective number of daughters of each bull. The covariance of **y** is **ZGZ** ^*T*^ + **R**, where $\mathbf{R}=\mathbf{D}{\sigma}_e^2$ and $\mathbf{G}=\mathbf{I}\frac{{\widehat{\sigma}}_a^2}{39557}$. The estimation of parameters of the above model was based on solving the mixed model equations: $$ \left[\begin{array}{c}\hfill \overset{\wedge }{\mathbf{b}}\hfill \\ {}\hfill \overset{\wedge }{\mathbf{g}}\hfill \end{array}\right]={\left[\begin{array}{cc}\hfill {\mathbf{X}}^T{\mathbf{R}}^{-1}\mathbf{X}\hfill & \hfill {\mathbf{X}}^T{\mathbf{R}}^{-1}\mathbf{Z}\hfill \\ {}\hfill {\mathbf{Z}}^T{\mathbf{R}}^{-1}\mathbf{X}\hfill & \hfill {\mathbf{Z}}^T{\mathbf{R}}^{-1}\mathbf{Z}+{\mathbf{G}}^{-1}\hfill \end{array}\right]}^{-1}\left[\begin{array}{c}\hfill {\mathbf{X}}^T{\mathbf{R}}^{-1}\mathbf{y}\hfill \\ {}\hfill {\mathbf{Z}}^T{\mathbf{R}}^{-1}\mathbf{y}\hfill \end{array}\right] $$ (Henderson 1984). The iteration on data technique was based on the Gauss–Seidel algorithm with residuals update (Legarra and Misztal 2008).

### Testing differences between populations

In order to test for differences in the variation of analyzed traits between both populations, genomic regions of various lengths were defined and SNP estimates were compared for German and Polish data within each region. In particular, each region *i* was defined as a window of a given length in base pairs and, for each such window, the null hypothesis assuming equality of SNP effects averaged for the region was tested against the alternative of differences in average effect using the two-sample *t*-test:

$$ {t}^i=\frac{{\overline{a}}_{DE}^i-{\overline{a}}_{PL}^i}{\sqrt{\frac{{\widehat{\sigma}}_{DE}^{2^i}+{\widehat{\sigma}}_{PL}^{2^i}}{N_{SNPi}}}} $$

where ${\overline{a}}_x^i$ represents the additive effect of the *i*th chromosome region estimated as the arithmetic mean of SNP effect estimates located within this region, respectively for the German (DE) and the Polish (PL) populations, ${\widehat{\sigma}}_x^{2^i}$ represents the variance of additive effects within the *i*th region estimated separately for each population, and *N*_*SNPi*_ is the number of SNPs within the *i*th region. The additive variance of the *j*th SNP within the *i*th region (${\widehat{a}}_x^{ij}$) was estimated as: ${\widehat{a}}_x^{ij}=2{\left({\widehat{g}}_x^{ij}\right)}^2{p}_x^j\left(1-{p}_x^j\right)$, where ${p}_x^j$ corresponds to the MAF of the SNP estimated for the base population following the approach of Gengler et al. (2007). The base population was defined here separately for the Polish and German populations as non-genotyped ancestors of the genotyped animals. In order to address the multiple testing problem, the false discovery rate (FDR) approach of Benjamini and Hochberg (1995) was applied based on the nominal *P*-values. The *t*-test and FDR correction were calculated using the SAS software. Since it is not possible to define an optimal window length arbitrarily, a selection of different lengths between 0.3 Mbp and 1.5 Mbp increasing by 0.1 Mbp was used.

## Estimation of SNP covariance between populations

The SNP genetic covariance between both populations was estimated using the following bivariate mixed model:

$$ \widehat{\mathbf{g}}=\mathbf{P}\boldsymbol{\upmu } +\mathbf{Zq}+\boldsymbol{\upvarepsilon} $$

where $\widehat{\mathbf{g}}$ is a vector of SNP effect estimates for the Polish and German populations, **μ** is a vector of population means, **q** is a random vector of SNPs, and **ε** is a vector of residuals. It is assumed that **q** ~ N(0, **I**_**q**_ ⊗ **G**) and $\boldsymbol{\upvarepsilon} \sim \mathrm{N}\left(0,{\mathbf{I}}_{\boldsymbol{\upvarepsilon}}{\upsigma}_{\boldsymbol{\upvarepsilon}}^2\right)$, where **I**_**q**_ and **I**_**ε**_ are identity matrices with dimensions corresponding to **q** and **ε**, respectively, **G** is a (co)variance matrix of SNP effect estimates between populations, and ${\sigma}_{\varepsilon}^2$ is a residual variance. **P** and **Z** are the corresponding incidence matrices. The ASreml software (version 3.0) was used for the estimation of model parameters and (co)variance components (Gilmour et al. 1995).

## Results

### Differences in linkage disequilibrium pattern

**Fig. 1:**
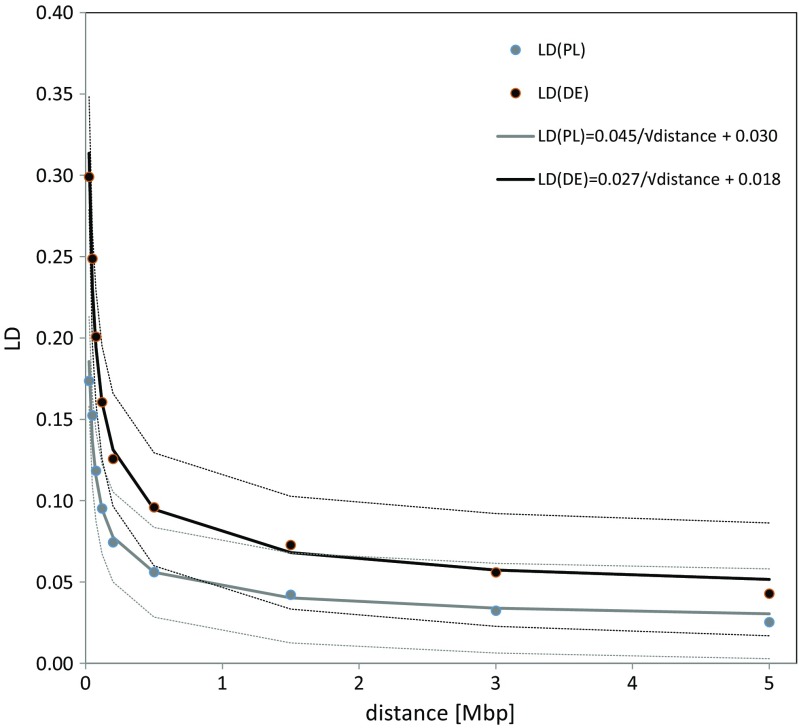
Empirical linkage disequilibrium (LD) decay curves and the corresponding regression functions estimated for the German and Polish populations, with 95% confidence intervals represented by *dashed lines*

At each distance defined in Mbp, a significantly lower LD was observed in the Polish than in the German population. Empirical curves and the corresponding regression functions for the Polish and German populations are visualized in Fig. 1. For each of the considered intervals, the average LD was significantly lower in the Polish than in the German population. However, with increasing genomic distance, LD decayed 1.7 times more rapidly (*P* = 5 × 10^−13^) in German than in Polish cattle. For instance, on a short scale between [0–0.025) and [0.05–0.075) Mbp intervals, LD was predicted to decrease, on average, by 0.08316 in the German population, but only by 0.04914 in the Polish population. Considering longer inter-marker intervals of [1.5–3) and [3–5] Mbp, the decrease was by 0.01073 in the German and by 0.00634 in the Polish breeds.

### Differences in effects of genomic regions

**Fig. 2:**
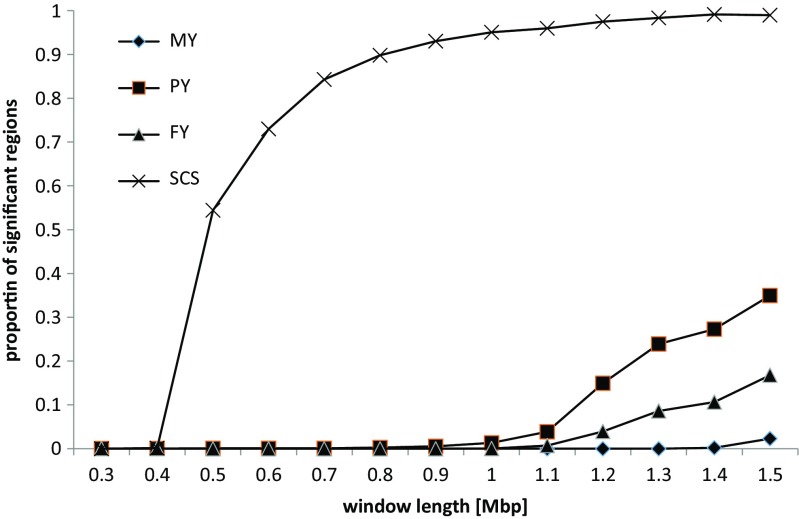
Proportion of significant regions for windows of different lengths

The comparison of genomic effects of chromosome regions was dependent on the length of the defined window. This situation made it impossible to identify and annotate regions responsible for population-wide differences. On the other hand, comparing results corresponding to different window lengths emerged a systematic pattern: regardless of the length of the compared regions between populations, only a few significant differences were found for production traits, while many were observed for SCS (Fig. 2). For example, windows of 0.6 Mbp in size split the bovine genome into 3819 regions, with an average number of 10.3 SNPs per region. No differences were identified for MY and FY, four regions were different for PY, but as many as 2787 regions were different for SCS, all with a maximum FDR of 10%.

### Differences in SNP allele frequencies in base populations

The comparison of SNP allele frequencies for base populations estimated separately based on Polish and German data revealed a very good agreement, expressed by high Pearson correlation coefficients of 0.97, and a correspondingly very good fit of a linear regression with the estimated intercept of 0.01, slope of 0.99, and the *r* ^2^ coefficient amounting to 0.95.

### Covariance between populations

The pattern of estimated (co)variance components for additive SNP effects was consistent across traits. For each trait, the German population had much higher SNP variances than the Polish population, with the ratio varying between 1.8 for SCS and 2.8 for PY. Genetic covariance estimates were all positive, resulting in genetic correlations of 0.22, 0.24, 0.30, and 0.39 for MY, PY, FY, and SCS, respectively.

## Discussion

Our comparisons showed that, although both populations seem to have a very similar genetic background, revealed by the high similarity of allele frequencies in base populations, the differences in selection goals and pressure imposed on Polish and German Holstein-Friesian populations over generations caused a detectable degree of differentiation.

The emerging picture based on the panel of medium density SNPs exhibits LD extending over several hundreds of kilobases in both populations. This is generally consistent with the results of previous studies in cattle populations, which used a similar panel of ascertained SNPs (e.g., Banos and Coffey 2010; Qanbari et al. 2010, among others). A denser catalog of unascertained SNPs generated from whole genome re-sequencing however, revealed LD decaying at a much faster rate in cattle (Qanbari et al. 2014). Given that the magnitude of LD as measured by *r* ^2^ depends on allele frequencies, the difference between the studies can be partially attributed to the biased SNPs selection on the genotyping arrays, where SNPs are ascertained non-randomly, aiming at frequent alleles and a comprehensive coverage of the genome, resulting in a uniform allele frequency spectrum. Furthermore, the assembly of large LD blocks that appeared in array-based analyses breaks into series of shorter tracts when LD is assessed from sequence data (e.g., Service et al. 2006). For further discussion on the comparison of array- vs. sequence-based LD, we refer to Qanbari et al. (2014). The faster LD decay observed in the German than in the Polish population is a consequence of a higher LD on average in that population, which is then diminished by recombination between SNPs, located further apart from one another. The strength of LD is of key importance for the genome-based analysis of population history, as it is indicative of genetic forces that a population has experienced during evolution, domestication, and selection.

The comparison of effects of genomic regions showed that, for production traits (MY, PY, and FY), the additive genetic effects are very similar in the German and Polish populations, especially for windows up to 1 Mbps in length, while an opposite picture emerges for SCS. This is supported by differences in selection programs between both countries. Production traits have long been in the focus of both Poland and Germany, indicating a similar selection pressure over generations. On the contrary, SCS has been included in the German total merit index already from 1997, while in Poland, the selection index has been enhanced by SCS only relatively recently, in 2008. This results in a strong variation pattern between the two analyzed populations.

Yet another aspect arises in the comparison of estimated genetic correlations between Poland and Germany. Polygenic-based genetic correlations published by Interbull (http://www.interbull.org/ib/maceev_archive, release December 2016) report lower estimates for production (0.84–0.90) traits than for SCS (0.96), which is in agreement with SNP-based correlations estimated in our study. Both models assume a normal distribution of genetic variation across the genome, but, in reality, for production traits, there is an accumulation of high effect SNPs (most remarkably the DGAT1 region on chromosome 14 with effects on MY and FY), which results in lower estimated country correlations despite no significant differences observed between particular genomic regions. In contrast to that, the inheritance mode of SCS appears to be of a purely polygenic nature, i.e., determined by many genes of moderate to small effects, and, thus, meets model assumptions, which is then reflected by a higher country correlation of 0.96. Note that, generally, lower SNP-based (this study) than polygenic-based (Interbull) correlations are due to no common individuals between the German and Polish pedigrees used in this study.

## Conclusions

The comparison of Polish and German Holstein-Friesian populations showed that observed differences in the estimated effects of genomic regions depend on differences in the linkage disequilibrium (LD) pattern between populations and on traits’ inheritance mode. Accounting for such differences has direct implications on the prospective international genomic evaluation based on across-country single nucleotide polymorphism (SNP) effect estimation. Therefore, a proposed option would be the use of a cumulated/averaged effect of SNP groups binned by their genomic location (bp) or, preferentially, by LD, instead of single SNP estimates in the SNP MACE model.
